# The Role of Cytokines and Molecular Pathways in Lung Fibrosis Following SARS-CoV-2 Infection: A Physiopathologic (Re)view

**DOI:** 10.3390/biomedicines12030639

**Published:** 2024-03-13

**Authors:** Mihai Lazar, Mihai Sandulescu, Ecaterina Constanta Barbu, Cristina Emilia Chitu-Tisu, Darie Ioan Andreescu, Andreea Nicoleta Anton, Teodora Maria Erculescu, Alexandru Mihai Petre, George Theodor Duca, Vladimir Simion, Isabela Felicia Padiu, Cosmina Georgiana Pacurar, Ruxandra Rosca, Teodor Mihai Simian, Constantin Adrian Oprea, Daniela Adriana Ion

**Affiliations:** 1 Faculty of Medicine, University of Medicine and Pharmacy Carol Davila, No. 37, Dionisie Lupu Street, Sector 2, 020021 Bucharest, Romania; mihai.i.lazar@gmail.com (M.L.); cristinaemilia_tisu@yahoo.com (C.E.C.-T.); darie-ioan.andreescu0720@stud.umfcd.ro (D.I.A.); andreeanicoleta.anton0720@stud.umfcd.ro (A.N.A.); teodora-maria.erculescu0720@stud.umfcd.ro (T.M.E.); alexandru-mihai.petre0720@stud.umfcd.ro (A.M.P.); george-theodor.duca0720@stud.umfcd.ro (G.T.D.); vladimir.simion0720@stud.umfcd.ro (V.S.); isabela-felicia.padiu0720@stud.umfcd.ro (I.F.P.); cosmina-georgiana.pacurar0720@stud.umfcd.ro (C.G.P.); ruxandra-raluca.rosca0720@stud.umfcd.ro (R.R.); teodor-mihai.simian0720@stud.umfcd.ro (T.M.S.); constantin-adrian.oprea0720@stud.umfcd.ro (C.A.O.); daniela.ion@umfcd.ro (D.A.I.); 2 National Institute for Infectious Diseases “Prof. Dr. Matei Bals”, No. 1, Calistrat Grozovici Street, Sector 2, 021105 Bucharest, Romania

**Keywords:** lung fibrosis, fibrosis mediators, fibrosis predictors, COVID-19, SARS-CoV-2 infection, long COVID-19

## Abstract

SARS-CoV-2 infection is a significant health concern that needs to be addressed not only during the initial phase of infection but also after hospitalization. This is the consequence of the various pathologies associated with long COVID-19, which are still being studied and researched. Lung fibrosis is an important complication after COVID-19, found in up to 71% of patients after discharge. Our research is based on scientific articles indexed in PubMed; in the selection process, we used the following keywords: “lung fibrosis”, “fibrosis mediators”, “fibrosis predictors”, “COVID-19”, “SARS-CoV-2 infection”, and “long COVID-19”. In this narrative review, we aimed to discuss the current understanding of the mechanisms of initiation and progression of post-COVID-19 lung fibrosis (PC-19-LF) and the risk factors for its occurrence. The pathogenesis of pulmonary fibrosis involves various mediators such as TGF-β, legumain, osteopontin, IL-4, IL-6, IL-13, IL-17, TNF-α, Gal-1, Gal-3, PDGF, and FGFR-1. The key cellular effectors involved in COVID-19 lung fibrosis are macrophages, epithelial alveolar cells, neutrophils, and fibroblasts. The main fibrosis pathways in SARS-CoV-2 infection include hypoxemia-induced fibrosis, macrophage-induced fibrosis, and viral-fibroblast interaction-induced fibrosis.

## 1. Introduction

Lung fibrosis follows an excessive and persistent increase in extracellular matrix deposition, inducing gas exchange impairment and hypoxemia. The lung fibrosis process is the final result of a complex homeostatic alteration involving inflammation, oxidative stress, chemoattractant mediators, and coagulation abnormalities, with cytokines such as TGF-β1, PDGF, IL6, IL11, and IL17 driving the underlying pro-inflammatory and profibrotic mechanisms [1]. The abnormal activation of TGF-β signaling induces the transcription of fibrotic genes (such as *SNAI1*, *SLUG*, and *Zeb1*), thus affecting multiple pathways implicated in fibrogenesis, such as myofibroblast proliferation or EMT (epithelial–mesenchymal transition) activation to promote fibrosis [2,3,4]. PDGF signaling plays a role in regulating fibroblast proliferation and differentiation, contributing to the production and deposition of collagen [2,3,4]. PDGF also enhances the release of TGF-β from activated macrophages and epithelial cells. IL-6 (known as both a pro-inflammatory and anti-inflammatory cytokine) is one of the cytokines responsible for activating M2-like macrophages with hyper-profibrotic phenotypes; the accumulation of these hyper-profibrotic cells follows, finally inducing extracellular matrix deposition and aggravating pulmonary fibrosis [2,3,4]. In addition, IL-11 is responsible for the differentiation of fibroblasts, thus resulting in collagen synthesis. IL-17, also a pro-inflammatory cytokine, is proven to determine EMT of alveolar type II cells, inducing a pro-fibrotic effect and inhibiting autophagy of alveolar epithelial cells in lung injury to promote fibrosis [2,3,4].

The excessive accumulation of fibrotic tissue within the lung parenchyma is a common feature of a large group of lung pathologies known as interstitial lung diseases (ILD). ILD include lung inflammatory pathologies, granulomatous diseases such as sarcoidosis and hypersensitivity pneumonitis, smoking-associated pathologies like respiratory bronchiolitis and desquamative interstitial pneumonitis, idiopathic pulmonary fibrosis (IPF), nonspecific interstitial pneumonitis, pneumoconioses, connective tissue diseases, and other systemic pathologies [5].

While IPF is the most prevalent ILD [6], there is a growing concern regarding the rising number and severity of lung inflammatory conditions.

Post-acute viral pulmonary fibrosis has been described in viral infections such as Influenza A (H1N1), HIV (human immunodeficiency virus), and Epstein–Barr virus (EBV). Influenza A (H1N1) virus can rapidly progress to acute respiratory distress syndrome (ARDS) and may induce pulmonary fibrosis. However, some studies suggest that this type of pulmonary fibrosis has the potential to undergo self-rehabilitation, which indicates that the underlying mechanism of this condition may differ from other lung diseases associated with pulmonary fibrosis. Studies have found fibrosis in different spatial distributions and sometimes with a delayed onset. A prevalence rate of 10% for post-ARDS pulmonary fibrosis has been reported [6]. Additionally, active cytomegalovirus infection has been associated with pulmonary fibrosis based on chest high-resolution computed tomography. Experimental studies have demonstrated that cytomegalovirus can accelerate existing fibrosis by enhancing TGF-β1 activation and increasing vimentin levels [7,8]. It has been found that pulmonary fibrosis affects 29.4% of HIV patients who have interstitial lung diseases, as per high-resolution computerized tomography. The presence of these changes has a significant positive correlation with HIV viral load [9]. Although there is still controversy surrounding the topic, some studies have indicated that Epstein–Barr virus (EBV) may be present in patients suffering from pulmonary fibrosis. In fact, around 40% of patients’ lung biopsies have shown the presence of EBV [10].

The COVID-19 pandemic began in 2019, and the virus responsible for it, known as SARS-CoV-2, is still a significant public health concern. This is due to the challenges posed by the acute infection as well as the various pathologies associated with long COVID-19 syndrome, which occurs after hospital discharge. The medical community has only recently started documenting and investigating this syndrome. COVID-19 is a multisystemic disease that evolves with short-term effects such as dysregulated immune responses, pulmonary or multiorgan failure, coagulopathy, and vascular involvement. It also has long-term effects such as pulmonary fibrosis, shortness of breath, chronic cough, extreme tiredness, sleep disorders, brain fog, myalgia, and headaches, especially in elderly men and patients with underlying chronic diseases [11,12]. Although pulmonary fibrosis following pneumonia is typically self-limiting and reversible, lung fibrosis following COVID-19 is a significant complication, affecting up to 71% of patients after discharge [13]. Many medical centers have reported pulmonary fibrosis as a long-term complication of COVID-19. According to a meta-analysis that included 2000 patients, the prevalence of post-COVID-19 pulmonary fibrosis is 44.9%, with higher rates among males (53.8% vs. 46.2%) and patients with a history of chronic obstructive pulmonary disease [14]. A study conducted by Huang et al. regarding long-term pulmonary complications among COVID-19 survivors revealed that 52.6% of the study population had abnormalities on high-resolution computed tomography six months post-COVID-19. The percentage was even higher, at 57%, for the patients who suffered severe COVID-19 and required intensive medical support such as a high-flow nasal cannula, noninvasive ventilation (NIV), or invasive mechanical ventilation (IMV) [15].

There is currently a lack of understanding about the molecular mechanisms of initiation and progression of post-COVID-19 lung fibrosis (PC-19-LF). In addition, there is a need to identify prognostic markers and risk factors associated with PC-19-LF. To address these gaps, this narrative review aims to highlight the cytokines, molecular mechanisms, and pathophysiological pathways involved in PC-19-LF, as well as the risk factors associated with its occurrence.

## 2. Materials and Methods

Our narrative review is based on scientific articles indexed in PubMed, including English language articles found in the search process, from 2020 to 2023. The following keywords were used in the screening process: “lung fibrosis”, “fibrosis mediators”, “COVID-19”, “SARS-CoV-2 infection”, and “long COVID-19”.

**Figure 1:**
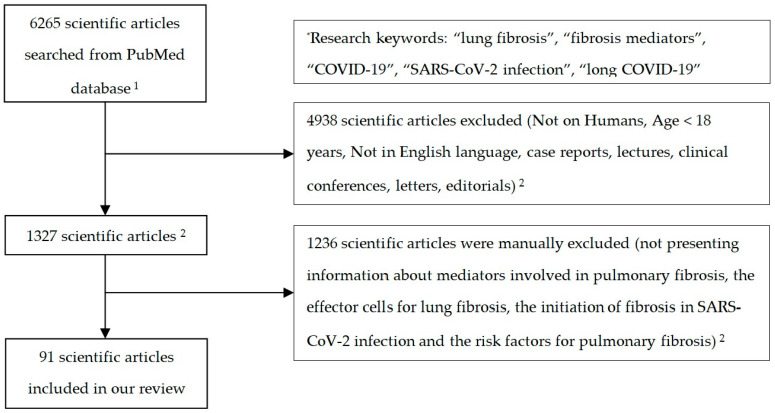
The flowchart of the review manuscript. Abbreviations:^1^ from 2020 till 2023; ^2^ in case of unclear or contradictory information, the supervisors of our review (ML and DAI) were consulted.

We excluded all case reports and studies with participants under 18 years old. We selected articles presenting information regarding the mediators involved in pulmonary fibrosis, the effector cells for lung fibrosis, the initiation of fibrosis in SARS-CoV-2 infection, and the risk factors for pulmonary fibrosis (Figure 1).

To ensure the gathering of comprehensive information for our review, we initially searched the titles and abstracts. If the available information was not sufficient, we performed a full-text documentation of the article. In case of unclear or contradictory information, the supervisors of the review (M.L. and D.A.I.) were consulted. Following the strategy presented above, we identified relevant articles to include in our review.

Graphical representations were created using www.biorender.com, accessed on 15 February 2024.

## 3. The Pathophysiology of Pulmonary Fibrosis

PC-19-LF represents a new long-term manifestation of COVID-19 whose definition, pathogenesis, diagnosis, and management are not yet fully understood.

### 3.1. Mediators Involved in Pulmonary Fibrosis

The mediators hypothesized to be involved in the pathogenesis of pulmonary fibrosis have a broad spectrum, being classified as cytokines, growth factors, or enzymes synthetized by various subtypes of macrophages or lymphocytes. These mediators ultimately induce the mechanisms considered essential for collagen synthesis by fibroblasts and myofibroblasts.

Wendisch D et al. studied the role of a certain macrophage population as a profibrotic trigger in SARS-CoV-2-infected patients [16]. These macrophages expressed a CD163+ phenotype and genes involved in the synthesis of TGF-β, osteopontin, and legumain [1].

When the balance between the production and clearance of lipidic components of the surfactant is disrupted, alveolar macrophages accumulate these lipids in an oxidized form within their cytoplasm and become “foam cells” [17]. The histopathological analysis identified these cells in the lungs of patients affected by numerous fibrotic pulmonary diseases, which led to the discovery that “foam cells” synthesize increased concentrations of TGF-β as a mechanism of pulmonary fibrosis [17].

TGF-β inhibits apoptosis and stimulates the proliferation of fibroblastic and myofibroblastic populations by activating the TGF-β/Smad signaling pathway [18]. Components of the extracellular matrix, such as integrin α_v_β_6_ and thrombospondin, are essential for the activation of the latent form of TGFβ [19]. The signal transducer and activator of transcription 3 (STAT3) coordinate this process by influencing the composition of the extracellular matrix [17]. TGF-β increases the production of collagen in fibroblasts and inhibits matrix metalloproteinase (MMP) 14, controlling the turnover of collagen [16]. A significant correlation has been found between plasmatic levels of TGF-β and the occurrence of pulmonary fibrosis after SARS-CoV-2 infection [20].

Legumain acts as a mediator of MMPs activity, influencing the extracellular matrix composition, and facilitates the transduction of profibrotic signals initiated by TGF-β; its function as an asparaginyl endopeptidase is prominent in SARS-CoV-2 infections [16].

*SPP1* gene encodes osteopontin, which, besides its structural role as a component of the extracellular matrix, can function as a cytokine; this type of cytokine is implicated in the synthesis of collagen I synthesis secondary to the activation of fibroblasts [16].

Interleukin 4 (IL-4), a T helper 2 cytokine, has overlapping activities with IL-13 due to its particularity of sharing a common receptor subunit, ultimately activating the JAK/STAT6 pathway. Both IL-4 and IL-13 levels were increased in patients with respiratory distress in the context of SARS-CoV-2 infection, confirmed by RT-PCR, in comparison with healthy individuals [21]. However, IL-13 influences the progression and severity of the disease mainly through a distinct mechanism of facilitating the accumulation of hyaluronan polysaccharide in the lungs rather than through the Th2 pathway [22]. Moreover, both IL-4 and IL-13 exhibit a role in the alternative activation of macrophages by stimulating the expression of Arg1, a marker often utilized for identifying alternatively activated macrophages [22,23]. Polarization of macrophages may result in either classically activated macrophages, which promote inflammation, or alternatively activated macrophages (AAM), involved in wound resolution and repair [24]. AAM were demonstrated to contribute to pulmonary fibrosis [25]. Following anti-IL13 treatment in patients with severe COVID-19, Donlan et al. discovered that Arg1 expression was substantially downregulated, further suggesting the involvement of IL-13 in the pathogenesis of pulmonary fibrosis secondary to COVID-19 infection [22]. However, it is important to note that some patients may benefit from IL-13 treatment, as Bonser et al. concluded that treating human bronchial epithelial cells with IL-13 may reduce COVID-19 viral RNA in these cells [26]. Thereby, supplementary studies are required in order to understand the role of IL-13 in pulmonary fibrosis induced by COVID-19 infection.

IL-6 can act as a pro-inflammatory stimulus, as well as an inducer of an anti-inflammatory response, by influencing the differentiation of macrophages [24]. IL-6 facilitates succeeding phases of the polarization process initiated by IL-4 and IL-13 by acting upon macrophages with increased expression of Arg1, Ym1, and CD206 [21]. IL-6 phosphorylates STAT3, and the signal is transduced in the nucleus, where profibrotic gene transcription is stimulated [18]. SARS-CoV-2 leads to the activation of pulmonary myofibroblasts through pathways such as IL-6/STAT3 or TGF-β/Smad which stimulate the upregulation of the *COL1A1* gene (encoding for collagen type I alpha 1 chain) in these cells [27]. Furthermore, SARS-CoV-2 can create an imbalance between the STAT1 and STAT3 signaling pathways by inhibiting STAT1 while hyperactivating STAT3 [28]. Additionally, STAT 1 inhibition is associated with epidermal growth factor receptor (EGFR) overexpression, which leads to STAT3 supplementary activation [28]. However, the relationship between EGFR and pulmonary fibrosis was evaluated as statistically insignificant by another author [20].

TNF-α, a cytokine with both pro-inflammatory and anti-inflammatory properties, can exist under two forms: transmembrane and soluble [20]. Profibrotic effects are mediated through the TNFR (tumor necrosis factor receptor) 1 receptor by regulating TGF-β1 expression [20]. Proudfoot et al. demonstrated that anti-TNFR1 antibodies may reduce pulmonary inflammation and endothelial injury, thus suggesting that TNF-α might play a role in lung disease [29]. In opposition, TNFR2, the other receptor of TNF-α, reduced hyperinflammation in COVID-19 acute respiratory distress syndrome (ARDS) after administering umbilical cord-derived mesenchymal stem cells [30]. A study performed by Maranatha et al. demonstrated a statistically significant correlation (*p* = 0.046) between TNF-α serum levels, which were increased in patients with COVID-19 and the pulmonary fibrosis developed after the infection [31].

IL-17 is a key inflammatory cytokine in different pulmonary diseases, including asthma, pneumonitis, and pulmonary fibrosis [31]. COVID-19 patients have also presented high levels of IL 17 [32]. The IL-17 family has six members: IL-17 A, IL-17 B, IL-17 C, IL-17 D, IL-17 E, and IL-17 F. IL-17 A was the first to be discovered and shares 55% sequence homology with IL-17 F [33]. They are produced by multiple cell types such as Th17, CD8+T cytotoxic 17, γδT17, macrophages, NK cells, neutrophils, and non-hematopoietic cells such as epithelial and endothelial cells [34]. By binding to corresponding receptors, IL-17 A and IL-17 F cytokines activate signaling cascades that ultimately lead to the progression of pulmonary fibrosis [35]. IL-17 A is involved in several processes that promote fibrosis, such as EMT, fibroblast proliferation, and transdifferentiation to myofibroblasts. IL-17 A also increases the levels of TGF-β1, a very important pro-fibrogenic mediator [36]. Moreover, IL-17 A stimulates neutrophil degranulation and oxidative stress damage, leading to the development of lung fibrosis [37]. IL-17 F is involved in human chronic tissue inflammation [38].

Galectins (Gal) are protein molecules that bind to sugar residues, specifically beta-galactoside sugars. They have several functions, including interactions between cells, adhesion, and transmembrane signaling. These molecules are also associated with different diseases, such as cancer and HIV. Recent studies have shown a link between galectins and post-COVID-19 pulmonary complications. They contribute to lung tissue remodeling and pulmonary fibrosis after COVID-19. Some studies have also proposed the use of galectin inhibitors as an effective treatment against PC-19-LF [39].

Galectin 1 (Gal-1) is one of the human galectins involved in the progression of idiopathic pulmonary fibrosis. Under hypoxic conditions, Gal-1 activates focal adhesion kinase 1 (FAK 1) in lung epithelial cells. FAK 1 mediates the transdifferentiation of fibroblasts into myofibroblasts, leading to extracellular matrix deposition, also known as lung fibrotic damage [40].

Galectin 3 (Gal-3) is the most studied galectin in terms of involvement in COVID-19 pathophysiology, complications, and treatment. Gal-3 is involved in the inflammatory response and tissue repair after the lung damage caused by SARS-CoV-2 [39]. This molecule is expressed in endothelial cells, fibroblasts, and resident alveolar macrophages. Lung fibrosis may be promoted by Gal-3 through different mechanisms. First of all, Gal-3 has an important role in the immune response and inflammation, leading to a cytokine storm syndrome prior to the development of lung fibrotic damage [41]. Moreover, Gal-3 promotes the TGF-β1 signaling pathway, enhancing EMT, extracellular matrix production, and alveolar epithelial cells apoptosis [42]. Gal-3 can also induce lung fibrogenesis by binding to and activating Toll-like receptor 4 (TLR4) [43]. In hypoxic conditions, pulmonary fibrosis can be triggered if Gal-3 binds to the triggering receptor expressed on myeloid cells 2 (TREM2) and activates it [44].

Platelet-derived growth factor (PDGF) is another important factor, exerting mitogenic, motogenic, and chemoattractant effects on fibroblasts [45]. PDGF signaling plays a role in regulating fibroblast proliferation and differentiation, as well as promoting the release of TGF-β from activated macrophages and epithelial cells. This contributes to a self-amplifying cycle involving collagen production and deposition.

Additionally, fibroblast growth factor receptors-1 (FGFR-1) are essential contributors to collagen synthesis and deposition and further activation of fibroblasts. In patients with idiopathic pulmonary fibrosis, FGF1-FGFR signaling may contribute to the pathogenesis of lung fibrosis by supporting fibroblast migration and increased MAPK signaling [46].

The mediators involved in PC-19-LF are summarized in Table 1.

### 3.2. Cellular Efectors in COVID-19 Lung Fibrosis

#### 3.2.1. Alveolar Macrophages

Grant et al. [47] analyzed the broncho-alveolar fluid (BAF) of mechanically ventilated patients with SARS-CoV-2 and found that the BAF contains a significant amount of T cells (CD4+, CD8+) and monocytes [48]. In only 31% of the patients, BAF neutrophilia was present. Moreover, as opposed to other pneumonia BAF samples, the BAF samples in SARS-CoV-2 pneumonia showed persistently elevated T cell levels, associated with high values of IFN γ, for both CD4+ and CD8+ T cells found in the BAF of COVID-19 patients [47]. Alveolar macrophages respond to the IFN-γ produced by T cells, with increased clustering being found in the BAF of COVID-19 patients [47]. Once activated, the alveolar macrophages will present the antigens to pre-existing cross-reactive memory T cells (elevated in the elderly [49]) and will increase the transcription of chemokines that attract T cells [48], creating an activation loop between macrophages and T cells. The activation of monocytes to monocyte-driven alveolar macrophages (MoAMs) playing an important part in lung fibrosis because alveolar macrophages, especially MoAMs, express elevated levels of profibrotic genes such as *CD163, MERTK* (Tyrosine-protein kinase Mer), *LGMN* (Legumain), *MMP9, TGF-β, TGF-β1, NRP1*, and *MRC1* (Mannose Receptor C-Type 1) [16]. Wendisch D et al. also demonstrated strong interactions between CD163+ macrophages (frequently found in SARS-CoV-2 infection) and mesenchymal cells such as fibroblasts, myofibroblasts, smooth muscle cells, and pericytes, thus promoting lung fibrosis [16].

An important aspect to consider in this process is that alveolar macrophages can also be infected directly or secondary to phagocytosis of an infected alveolar epithelial cell, followed by the escape of the virus from the lysosome; therefore, alveolar macrophages may serve as a viral pool for SARS-CoV-2 and can support its replication [47].

#### 3.2.2. Endothelial to Mesenchymal Transition

Endothelial to mesenchymal transition (EndMT) is mediated by TGF-β using pathways such as SMAD, MEK, PI3K, and p38 MAPK [50]. This transition can further generate fibroblasts [51]. It is shown that EndMT contributes to fibrotic diseases, such as cardiac fibrosis [52,53] and it was more recently proven that EndMT is present in idiopathic pulmonary fibrosis [54]. Thus, the high levels of TGF-β found in COVID-19 patients may contribute, through the EndMT pathways, to pulmonary fibrosis [54].

#### 3.2.3. Epithelial Alveolar Cells

Epithelial alveolar cells type I and II play an important role in lung fibrosis, especially type II cells. They initiate lung inflammation that leads to fibrosis, and they also are key components of tissue remodeling through re-epithelization processes [55].

SARS-CoV-2 induces necroptosis in epithelial alveolar cells (both type I and type II) by multiple pathways: increased TNF and caspase 8 concentrations promote caspase 3 dependent apoptosis [55] and higher programmed cell death gene expression like STAT1 [56]. Additionally, infection induces loss of epithelial junction integrity (downregulation of apical and tight junctions and claudins), which leads to marked alveolar cell desquamation and also disrupts the alveolar basal membrane, which represents the basis for marked alveolar tissue architecture changes characterized by fibrosis and impaired re-epithelization [55]. Another important change in the initiation of fibrosis is the induction of a senescence state in the AT2 cells (evidenced by an increased p21 and p16 nuclear expression), which makes them produce a large number of pro-inflammatory molecules, most importantly, TGF-β and IL-8 (important for its interaction with neutrophils) [55,57].

Another important mention is the presence of ectopically epithelial tuft-like cells in the lung parenchyma of patients infected with the COVID-19 virus, which may promote chemotaxis of macrophages and myeloid cells and would maintain inflammation with consecutive fibrosis and impaired alveolar regeneration [58].

#### 3.2.4. Neutrophils

Neutrophils play an important role in lung fibrosis in COVID-19 infection, mainly by the NETosis pathway (a programmed neutrophil cell death that can be activated by pro-inflammatory cytokines like IL1-β or IL-8) [57]. This pathway leads to the formation of NETs (neutrophil extracellular traps) that were demonstrated to stimulate EMT, which further promotes fibrosis [57]. This mechanism is evidenced by cells that are positive for both CK7 and α-SMA (α Smooth Muscle Actin), markers for epithelial and mesenchymal cells, respectively [56]. Experimental studies demonstrated that alveolar damage alone is not sufficient to initiate fibrosis; it also requires the presence of NETs [57,59]. Inflammatory cytokines (such as IL-17) stimulate neutrophil degranulation and increase oxidative stress damage, augmenting fibrotic changes [59].

#### 3.2.5. Fibroblasts

Pro-inflammatory cytokines (IL-6, TNF-α, and TGF-β) secreted by macrophages and T cells transdifferentiate fibroblasts into a specialized phenotype (myofibroblasts) that has an important role in wound closure [60] by synthesizing α-SMA and promoting irreversible contraction, with architectural changes and further production of the extracellular matrix (ECM) (Collagen I—mature/Collagen III—immature and fibronectin) [61].

Macrophages, bronchial epithelial cells, and type 2 pneumocytes highly express angiotensin-converting enzyme 2 (ACE2) receptors, leading to viral injury of the infected cells [62,63]. In COVID-19, the epithelial cells that populate the distal airways produce high quantities of mediators responsible for migration, growth, and activation of fibroblasts and their transformation into myofibroblasts, responsible for producing excessive and disordered surrounding ECM [45]. The dominant profibrotic factor is considered TGF-β, expressed by damaged epithelial, endothelial cells, macrophages, and the fibroblast itself after activation [64].

Activation of the PI3K/AKT/mTOR pathway reduces autophagy in fibroblasts and myofibroblasts while inhibiting EF2K and p38 MAPK signaling decreases autophagy processes, ultimately leading to reduced lung fibroblast apoptosis [65], increasing the fibrotic changes in COVID-19 patients.

SARS-CoV-2 presents a broad tropism for various receptors, including integrins α_v_β_3_ and α_v_β_6_. Integrin α_v_β_6_ fosters the transdifferentiation of fibroblasts into myofibroblasts and the EMT mediated by TGF-β1, thus triggering fibrogenesis when the virus is attached [66].

Another factor involved in COVID-19 lung fibrosis is represented by the mechanical traits of the laid-down ECM as the primary determinant in controlling myofibroblast function. Interaction with a more rigid matrix amplifies their synthesis activity, establishing a reinforcing loop [67].

A unique subtype of interstitial fibroblasts, pulmonary lipofibroblasts, are positioned in proximity to type 2 alveolar epithelial cells, serving the role of delivering triglycerides to these epithelial cells [68,69]. When exposed to different triggers (infection), pulmonary lipofibroblasts can shift from myofibroblasts and potentially add to pulmonary fibrosis [70]. Even though the exact process by which lipofibroblasts induce pulmonary fibrosis after SARS-CoV-2 infection is unclear, there seems to be a positive connection between the number of pulmonary lipofibroblasts and the severity of pulmonary fibrosis [71], particularly among diabetic or obese patients [72].

#### 3.2.6. The Response of Fibroblast to Specific Cytokines

Fibroblasts can be activated through their close interaction with the injured alveolar epithelium or the locally activated macrophage population (mediated by cadherin-11) or through interaction with specific cytokines (following epithelial/endothelial injury and activation of local immune response).

In pulmonary fibrosis, overexpression of TGF-β drives fibroblast proliferation, consequently transdifferentiation to contractile myofibroblast phenotype, and induces the production and deposition of ECM proteins. Three isoforms of TGF-β are known: TGF-β1, 2, and 3. In the pathogenesis of pulmonary fibrosis, TGF-β1 is predominantly expressed and plays a dominant role. Three TGF-β-receptors, namely I, II, and III, are found on the surface of cells, mediating its effect. TGF- β activates the release of cytokines such as PDGF, IL-1, basic FGF (bFGF), and TNF-α and automatically regulates its cascade [73,74]. The TGF-β1 isoform is considered responsible for the inhibition of ECM (extracellular matrix) degradation by matrix metalloproteinase, and there is also evidence of increased fibroblast growth factor (FGF)-2 alveolar secretions as a consequence of TGF-β1-upregulated expression. TGF-β exerts its profibrotic activity, stimulating several pathways, and one of the most relevant is the TGF-β Smad cascade, where activated TGF-β receptors are translocated to the nucleus by regulating other Smad proteins, leading to the phosphorylation of Smad-2 and Smad-3. Evidence was found that Smad-3 deficiency attenuates pulmonary fibrosis and that the inhibitor Smad-7 prevents the phosphorylation of Smad-2 and Smad-3, reducing the fibrotic phenomenon [75].

Profibrotic responses of fibroblasts are also enhanced by the release of growth factors from injured alveolar epithelium, such as PDGF, a potent mitogen for lung fibroblast and CTGF (connective tissue growth factor), which stimulates fibroblast proliferation and increases collagen production, further contributing to the development and progression of pulmonary fibrosis [76]. The fibroblast growth factor, produced by the activated local macrophage population, also contributes to lung fibrosis, increasing MAPK-signaling, fibroblast migration, activation, and collagen synthesis [46].

Many inflammatory cytokines released by the injured alveolar epithelium have a major impact on fibroblast pro-fibrotic response. IL6 was proven as a mitogen for fibroblasts in fibrotic lung tissue. Additionally, Wnt1-inducible signaling protein 1 (WISP1)-induced fibroblast proliferation is mediated by IL6; IL6 stimulates the phosphorylation of STAT3, followed by the activation of profibrotic genes [77].

The fibroblast response to interleukin-25 (IL25)-enhanced secretion is their proliferation and augmentation of collagen production. Moreover, Th17 cytokine interleukin-17 (IL17) and Interleukin-11 (IL11, through an extracellular signal-regulated kinase (ERK)-dependent pathway) were also proven to increase fibroblast proliferation, transformation into myofibroblasts, and collagen production [78].

**Figure 2:**
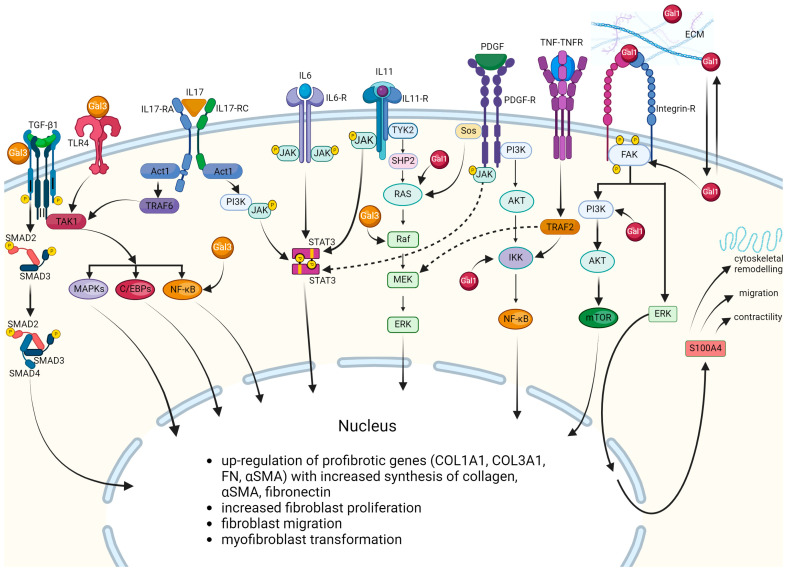
Fibroblast–cytokine interactions in SARS-CoV-2 infection. Abbreviations: Act1–adaptor protein Act1, AKT—alpha serine/threonine-protein kinase, EBPs—enhancer-binding proteins, ECM—extracellular matrix, ERK—extracellular signal-regulated protein kinase, FAK—focal adhesion kinase 1, FN—fibronectine, IKK—IkappaB kinase, IL—interleukin, IL6-R—receptor for interleukin 6, IL17-R—receptor for interleukin 17, IL11-R—receptor for interleukin 11, Gal—galectin, JAK/STAT—Janus kinase/signal transducer and activator of transcription, MAPK/MEK—mitogen-activated protein kinases, mTOR—*mechanistic target of rapamycin*, NF-κB—nuclear factor kappa-light-chain-enhancer, PDGF—platelet derived growth factor, PI3K—phosphoinositide 3-kinase, RAF—rapidly accelerated fibrosarcoma kinase, SHP2—Src homology 2 domain-containing *protein tyrosine phosphatase,* α-SMA—α smooth muscle actin, SMAD—mothers against decapentaplegic homolog 1, STAT3—*signal transducer and activator of transcription 3*, TAK—transforming growth factor beta activated kinase, TGF-β—transforming growth factor beta, TLR—Toll-like receptor, TNF α—tumor necrosis factor α, tumor necrosis factor α receptor—TNFR, TRAF—tumor necrosis factor receptor-associated factor, and TYK-tyrosine kinase.

The response of fibroblasts to specific cytokines in SARS-CoV-2 infection is represented in Figure 2.

Cellular effectors and their roles in PC-19-LF are summarized in Table 2.

### 3.3. Fibrosis Pathways in SARS-CoV-2 Infection

#### 3.3.1. Hypoxemia-Induced Fibrosis

The lung inflammation caused by SARS-CoV-2 leads to diffuse alveolar damage, alveolar–capillary barrier dysfunction, edema, alveolar exudate containing neutrophils and macrophages, reduced surfactant synthesis because of loss of type II alveolar epithelial cells (type II pneumocytes), fibrin deposition and microthrombi, formation of hyaline membranes, thickening, and congestion of alveolar septa filled with lymphomonocytic infiltrate and fibroblasts, intra-alveolar epithelial debris due to hyperplastic pneumocytes desquamation, and interstitial fibrosis [55,79]. These histopathological modifications of the lungs result in diminished gas exchange, which causes hypoxemia [55]. Hypoxemia manifests mainly as oxygen saturation of 93% or less, dyspnea, and polypnea (≥30 breaths/min) [80], and it may evolve into ARDS in critical COVID-19 cases [55].

It is worth mentioning that COVID-19-associated ARDS cases manifest through “happy”/“silent” hypoxemia or the hypoxemia/hypocapnia syndrome, which refers to the presence of hypoxemia in the absence of dyspnea [81]. Among the possible theories explaining the physiopathology of silent hypoxemia were the alteration of the cortical center of breathing through its inflammation caused by the traveling of SARS-CoV-2 from the nasal cavity to the brain or the reduction in sensitivity of the carotid body when dealing with hypoxia due to the ACE2 receptors present there which connect to the virus [82]. Another hypothesis implies the cytokine storm that causes pulmonary neovascularization and, further, hypoxia through a right-to-left shunt; hypoxia compensatory ventilation causes hypocapnia, which then hinders the additional ventilation, preventing dyspnea [82]. The hypoxemia induced by COVID-19 is responsible for the increase in Gal-1, a lectin proven to be a major factor in pulmonary fibrosis, also considered a “hypoxia-responsive protein” [40]. Thus, the crucial role of hypoxia in hyperplastic type II alveolar epithelial cells was studied and it underlined the profibrotic activation of lung cells through the interaction of Gal-1 and FAK 1 [40]. Incessant hypoxia in type II alveolar epithelial cells determines the upregulation of hypoxia-inducible factor-1α (HIF-1α) and also hypoxia-inducible profibrotic genes, including TGF-β1 [40]. HIF-1α was proven to stimulate the production of inflammatory cytokines and the N protein of SARS-CoV-2, hence regulating viral replication [83]. TGF-β1 interplays with the profibrotic pathway Wnt/β-catenin, elevating phosphorylated FAK-1 (y-397) levels [40]. FAK-1 is also activated when Gal-1 is working with the *Wnt3* gene [40]. FAK-1 is a non-receptor tyrosine kinase [40] involved in lung fibrosis through the FAK/ERK/S100A4 signaling pathway [84], resulting in the cytoskeletal remodeling and fibroblast migration, proliferation, and differentiation into myofibroblasts; the emerging myofibroblasts start secreting high levels of collagen and α-smooth muscle actin (α-SMA) [15,31], substances which, in return, amplify the over-expression of FAK-1 [84].

Additionally, it was observed how hypoxia caused elevated expression of not only mRNA profibrotic genes for PDGF subunit B, TGF-β, TNF-α, endothelin-1 (EDN1), and plasminogen activator inhibitor-1 (PAI-1), but also of mRNA-derived ECM proteins, including collagen, fibronectin, and MMP [40].

**Figure 3:**
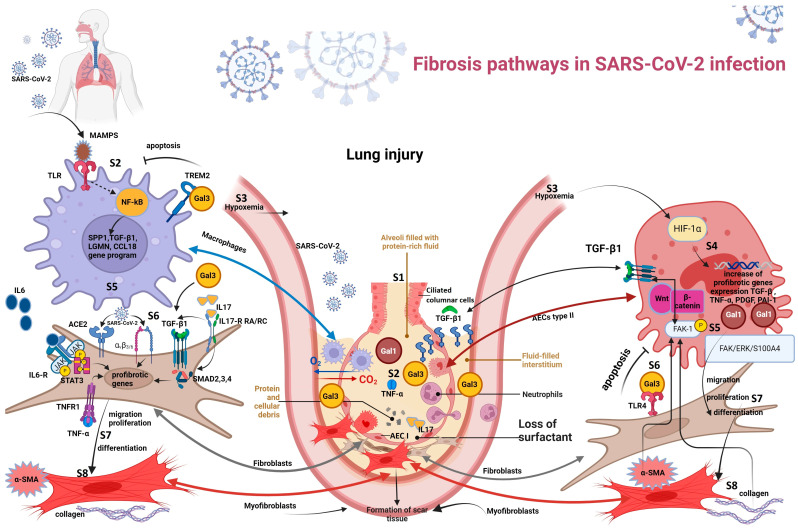
Fibrosis pathways in SARS-CoV-2 infection. Steps to lung fibrosis: S1. alveolar injury; S2. macrophages and neutrophils activation; S3. hypoxemia; S4. upregulation of HIF-1α and profibrotic genes; S5. production of inflammatory cytokines; S6. fibroblast–cytokine/SARS-CoV-2 interactions; S7. fibroblast migration, proliferation, and differentiation; S8. ECM changes (lung fibrosis). Abbreviations: AEC I—alveolar epithelial cell type I, AECs type II—alveolar epithelial cells type II, ECM—extracellular matrix, FAK 1—focal adhesion kinase 1, IL—interleukin, HIF-1α—hypoxia-inducible factor-1α, IL6-R—receptor for interleukin 6, IL17-R—receptor for interleukin 17, Gal—galectin, JAK/STAT—Janus kinase/signal transducer and activator of transcription, PDGF—platelet-derived growth factor, PAI—plasminogen activator inhibitor-1, MAMPS—microbe-associated molecular patterns, NF-κB—nuclear factor kappa-light-chain-enhancer, SMAD—mothers against decapentaplegic homolog 1, STAT3—signal transducer and activator of transcription 3, TGF-β—transforming growth factor beta, TLR—Toll-like receptor, TREM2—triggering receptor expressed on myeloid cells 2, TNF α—tumor necrosis factor α, tumor necrosis factor α receptor—TNFR, and α-SMA—α smooth muscle actin.

Fibrosis pathways in SARS-CoV-2 infection are represented in Figure 3.

SARS-CoV-2 infection induces an inflammatory phase and, depending on its intensity, it may progress to lung fibrosis [44]. When an excessive secretion of pro-inflammatory cytokines (including IL-1, IL-6, and TNF-α) driven by the adaptative immune response is involved, we talk about “the cytokine storm syndrome” [44]. The severity of the cytokine storm is correlated with the severity and mortality of COVID-19 [85]. Gal-3 has the capacity to induce cytokine storms and lung fibrosis after binding to the ACE receptor, similar in the structure of the extracellular domain with the ACE2 receptor, suggesting the possibility of an interaction between Gal-3 and ACE2, although it has not been yet concluded [44]. Furthermore, Gal-3 also binds to receptors such as CD147 and CD26 (whose implication in the Middle East respiratory syndrome coronavirus (MERS-CoV) epidemic was identified), which are suspected to interplay with the SARS-CoV-2 spike protein, contributing to the viral invasion and, further, to the elevated levels of inflammatory cytokines [44]. Gal-3 frequently ties to TREM2 and SPP1, two markers involved in fibrotic evolution, secreted by a subset of macrophages [86], but also to TLR4 [44]. TREM2 hinders macrophage apoptosis, leading to chronic lung inflammation, while the TLR family participates in the immune response against the viral infection, inducing the synthesis of pro-inflammatory factors [44]. Thus, Gal-3, TLR4, and TREM2 have a potentially major role in lung fibrosis post-viral infection [44]. The effect of TLR4 in pulmonary fibrosis via fibroblast activation has been recently emphasized, as well as its inhibition leading to an obvious fibrotic decrease [87]. After being confirmed that COVID-19 patients have higher levels of Gal-1, Gal-3, and prostaglandin E2 (PGE2) compared to healthy patients [39], the positive association between Gal-1 and IL-1β, IL-6, IL-10, IL-23, IL-33 was studied and proven [88]. COVID-19 patients also revealed a major increase in C-reactive protein (CRP), IL-2, IL-4, IL-6, IL-10, TNF-α, and IFN-γ [88]. IL-6 and IL-10 may serve as indicators for the early diagnosis of patients at risk of disease progression [85]. It appears that the level of IL-10 is directly proportional to Gal-1, especially in stage III of COVID-19 [76] and to CRP [83]. Hence, Gal-1 advocates IL-10’s immunosuppressive role on macrophages, T lymphocytes, and natural killer cells and promotes tissue injury while interfering with viral eradication [88]. CRP level is also positively correlated with Galectin-9 (Gal-9), a lectin proved to induce monocyte and NK cell production of IL-6 and TNF-α [89]. Moreover, a recent study underlined Gal-9’s capacity to increase the viral spike protein’s affinity to the type II alveolar epithelial cells, facilitating SARS-CoV-2’s entry [90]. The inflammation from COVID-19 can also culminate in pulmonary fibrosis via the NLRP3 inflammasome [80]. The NLRP3 inflammasome is activated by the viral entry in pneumocytes and their destruction, and it acts by fuelling the cytokine storm, elevating the levels of pro-inflammatory cytokines, such as TNF-α, IL-6, and IL-1β [80]. It was also observed how the SARS-CoV-2 binds to the macrophage ACE2 receptors, increasing IL-1 and IL-18 production and amplifying pyroptosis and lung hyperinflammation [91]. Additionally, it stimulates the neutrophilic infiltrate [80]. Thus, the NLRP3 inflammasome aggravates pulmonary tissue damage, favoring impaired gas exchange (leading to hypoxemia) and, ultimately, scarring of the tissue [80].

#### 3.3.2. Macrophage-Induced Fibrosis

In COVID-19, analyses of damaged lung tissue revealed an increase in macrophage density with a higher proportion of CD163 positive population. Furthermore, detailed analysis showed that CD163/LGMN-Mφ was one of the dominant populations of the recently recruited monocyte-derived macrophages during the first four weeks of COVID-19 ARDS, alongside FCN1-Mono and Mono-Mφ; SARS-CoV2 may induce CD163/LGMN-Mφ macrophage population phenotypes that strongly resemble the ones of IPF-specific macrophages [16]. CD163/LGMN-Mφ macrophages express genes with known involvement in the pathological sequela of fibrosis, such as SPP1, TGF-β I, LGMN, and CCL18. Moreover, the analysis showed a strong interaction between CD163/LGMN-Mφ and myofibroblasts, fibroblasts, and pericytes, implying important fibrotic pathways that involve Col, FGF (fibroblast growth factor), TGF-β1, and SPP1, among others. To a lesser extent, the same can be related to the Mono-Mφ population [12].

After being infected by SARS-CoV-2, an increase in TGF-β1 and CTGF mRNA transcripts was also demonstrated in alveolar epithelial cells, leading to lung fibrosis [92].

#### 3.3.3. Viral–Fibroblast Interaction

Norris et al. observed a direct binding of the receptor binding domain of the spike protein (S1-RBD) of SARS-CoV-2 to recombinant human αvβ3 and αvβ6 integrins. This binding initiates the processes of cell spreading, focal adhesion formation, and actin stress fiber organization, which are similar in extent to those caused by fibronectin. Additionally, S1-RBD stimulates the tyrosine phosphorylation of FAK, Src, and paxillin, triggers Akt activation, and supports cell proliferation. Thus, the RGD sequence of S1-RBD can act as an α_v_-selective integrin agonist [93]. This study also suggests that cell surface αv-containing integrins may respond functionally to spike protein, which raises the possibility that S1-mediated dysregulation of ECM dynamics could contribute to the pathogenesis of PC-19-LF [93].

SARS-CoV-2 can also induce fibrosis through its nucleocapsid (N) protein, shown to be induced in HFL-1 (human fibroblast line)-type cells with a heightened expression of α-SMA levels [94]; thus, these cells are inclined towards a more myofibroblast-like expression [95]. Myofibroblasts, due to their profibrotic phenotype, are known to respond to or secrete in abnormal fashion growth factors, other mediators, and ECM proteins (that includes enhanced collagen, TGF-β1, MMP-9, and tissue inhibitors of metalloproteinase (TIMP) expression) [96].

Moreover, when HFL-1 cells expressed N protein, there was a marked increase both in the expression of COL1A1 and in the secretion of collagen proteins, indicating a possible way in which SARS-CoV-2 could activate in a cell-autonomous manner in fibroblasts, potentially leading to lung fibrosis [94].

TGF-β1 promotes fibrosis through its role regarding fibroblast proliferation and differentiation into myofibroblasts and the subsequent secretion of ECM proteins by myofibroblasts. Moreover, TGF-β inhibits the action of MMP on ECM proteins [97].

In addition, the virus determined a rise in FN (fibronectin) 1 gene expression, with a previous study providing a link between lung fibrosis and increased FN deposition, thus indicating an incipient mean by which the virus might drive the development of lung fibrosis [98,99].

The specific aspects of PC-19-LF are presented in Table 3.

### 3.4. Risk Factors for Lung Fibrosis

The risk factors for PC-19-LF are related to COVID-19 severity and patient features. The elderly, smokers, diabetics, obese patients, those who need mechanical ventilation for severe acute respiratory distress syndrome, and those with hiperinflammation syndrome (high levels of pro-inflammatory cytokines, PCR, and high production of autoantibodies) have a higher risk of pulmonary involvement [100,101,102].

Additionally, patients with prior chronic interstitial lung diseases (ILD) present a high risk of developing parenchyma lesions as a complication after the acute phase of COVID-19 [103]. Patients who developed pulmonary fibrosis were shown to have an elevated inflammatory status sustained by inflammatory markers such as CRP or ESR [104]. Inflammation could lead to pulmonary fibrosis due to various and complex mechanisms detailed in Section 3.1, Section 3.2 and Section 3.3.

Lower plasmatic levels of IFN-γ, IFN-α2, and MCP-3 (2-, 1.3-, and, respectively, 1.3-fold lower), and higher plasmatic values of CRP (2.6-fold higher) were found in patients with fibrosis compared to those without fibrosis [105]. Furthermore, increased expression of mitochondrial biomarkers was found in patients with long-term pulmonary sequelae post-COVID-19, further indicating a correlation between oxidative stress induced by a higher inflammatory status and long-term pulmonary complications [106].

Higher fibrosis risk was communicated in patients with severe forms of COVID-19 (patients with ARDS) [107] and further increased in patients with mechanical ventilation [92]. Mechanical ventilation can produce stretch force injury, known as ventilator-induced lung injury, which can stimulate pro-inflammatory cytokine production and collagen forming [108]; mechanical injury may also be a stimulus for the secretion of TGF-β [109]. It was observed that ventilated patients with ARDS developed lung fibrosis after five days [108].

Hematological markers were also associated with the occurrence of pulmonary fibrosis; low erythrocytes, leukocytes, and thrombocytes values were observed in most patients with post-COVID-19 fibrosis [104], while elevated values for leukocytes (neutrophils) and thrombocytes were found in patients whose imaging findings showed an improvement over time [110]. Indeed, in one of our published studies, we found that the lower the lymphocytes, platelets, and hemoglobin, the higher the mortality for patients with severe COVID-19 forms [111].

D-dimers higher than 1 µg/mL were not only a predictive factor for a poor prognosis even from an early stage of the disease [112], but they were significantly associated with pulmonary fibrosis compared to patients with normal D-dimers values [104].

Pulmonary fibrosis represents a concern in the case of elderly patients (66 +/− 15 years) [13,103] who developed severe symptoms and spent a longer period of time in the hospital (24 days compared to only 19 spent by those in the control group) and have a delayed hospital admission by approximately 2 days [113]. Although a higher risk of developing pulmonary fibrosis was correlated with age and comorbidities (such as diabetes, lung, liver, and vascular diseases), there is evidence that suggests that lung fibrosis can appear in young, healthy individuals after SARS-CoV-2 infection [114], even in cases with mild symptoms and a short hospital stay, if the high value of virus RNA were persistent [115].

Longer intensive care unit (ICU) stay (over 7 days), mechanical ventilation, the severity of acute infection [116], and use of high-flow oxygen support (mean oxygen flow was 6+/− 4 L/min) [104] are also mentioned as risk factors for post-COVID-19 fibrosis.

Smoking increases oxidative stress and can generate systemic inflammation [117] and lung fibrosis [118], which are also independent factors. It can lead to a more severe infection [119] by inhibiting immune activity [117], which correlates with a higher need for ICU stay [116].

Chronic alcoholism is an oxidative stress factor, a pro-inflammatory factor, and decreases the glutathione level; these effects can complicate the evolution of an acute lung injury and increase the risk for interstitial fibrosis by inducing pulmonary secretion of TGF-β [120]. Chronic alcohol intake may also increase the need for ICU stay (due to hepatic injury), alter the immune system response, and increase the viral-induced inflammation [121].

## 4. Conclusions

The development of pulmonary fibrosis is influenced by various mediators, including cytokines, growth factors, and enzymes. Among these mediators, TGF-β is considered to be the most impactful molecule. Other important mediators associated with lung fibrosis are legumain, osteopontin, IL-4, IL-6, IL-13, IL-17, TNF-α, Gal-1, Gal-3, PDGF, and FGFR-1.

Macrophages, epithelial alveolar cells, neutrophils, and fibroblasts are the main cellular effectors involved in COVID-19 lung fibrosis.

The main fibrosis pathways in pneumonia, also found in SARS-CoV-2 infection, are hypoxemia-induced fibrosis and macrophage-induced fibrosis.

PC-19-LF has an additional pathway represented by viral–fibroblast interaction specific to COVID-19 patients.

A high risk of lung fibrosis should be considered in the elderly, smokers, chronic alcohol users, diabetics, and obese patients. Additionally, patients who require mechanical ventilation for severe acute respiratory distress syndrome, those with high D-dimers, hyperinflammation syndrome, or patients with prior chronic ILD are also at increased risk. Lower levels of IFN-γ, IFN-α2, MCP-3, erythrocytes, leukocytes, and thrombocytes in the plasma are also considered risk factors for PC-19-LF.

High persistent levels of virus RNA increase the risk of PC-19-LF, even in cases with mild symptoms, short hospital stays, or young, healthy individuals.

## Figures and Tables

**Table 1:** Mediators involved in pulmonary fibrosis.

Parameter	Mechanism Leading to Lung Fibrosis
TGF-β	inhibits apoptosis and stimulates the proliferation of fibroblastic and myofibroblastic populations by activating the TGF-β/Smad signaling pathway increases the production of collagen in fibroblasts and inhibits MMP 14 [18]
Legumain	an asparaginyl endopeptidase facilitates the transduction of profibrotic signals initiated by TGF-β induces changes in the extracellular matrix composition [16]
Osteopontin	activates fibroblasts, increasing collagen I synthesis [16]
IL-4	activates the JAK/STAT6 pathway activates macrophages by stimulating expression of Arg1 [21,22,23]
IL-6	facilitates succeeding phases of the polarization process initiated by IL-4 and IL-13 by acting upon the activated macrophages phosphorylates STAT3 and stimulates upregulation of the *COL1A1* gene (encoding for collagen type I alpha 1 chain) in pulmonary myofibroblasts [18,24,27,28]
IL-13	activates the JAK/STAT6 pathway activates macrophages by stimulating the expression of Arg1 facilitates the accumulation of hyaluronan polysaccharides in the lungs [19,20,21]
IL-17	activates signaling cascades that ultimately lead to the progression of pulmonary fibrosis [35] promotes fibrosis through EMT fibroblast proliferation and transdifferentiation to myofibroblasts [36] increases the levels of TGF-β1, an important pro-fibrogenic mediator [36] stimulates neutrophil degranulation and oxidative stress damage, leading to the development of lung fibrosis [37]
TNF-α	profibrotic effects are mediated through the TNFR 1 receptor by regulating TGF-β1 expression [20]
Gal-1	under hypoxic conditions, Gal-1 activates FAK 1 in lung epithelial cells. FAK 1 mediates the transdifferentiation of fibroblasts into myofibroblasts, leading to extracellular matrix deposition, also known as lung fibrotic damage [40]
Gal-3	promotes the TGF-β1 signaling pathway, enhancing EMT, extracellular matrix production, and alveolar epithelial cells apoptosis [42] induces lung fibrogenesis by binding to and activating TLR4 [43] in hypoxic conditions, pulmonary fibrosis can be triggered if Gal-3 binds to TREM2 (triggering receptor expressed on myeloid cells 2) and activates it [44]
PDGF	exerts mitogenic, motogenic, and chemoattractant effects on fibroblasts promotes the release of TGF-β from activated macrophages and epithelial cells [45]
FGFR-1	supports fibroblast migration and increased MAPK signaling [45].

Abbreviations: Arg1—arginase 1, COL1A1—collagen type I alpha 1, EMT—epithelial–mesenchymal transformation, FAK 1—focal adhesion kinase 1, FGFR—fibroblast growth factor receptor, IL—interleukine, Gal—galectin, JAK/STAT—Janus kinase/signal transducer and activator of transcription, MMP 14—matrix metalloproteinase 14, PDGF—platelet-derived growth factor, TGF-β—transforming growth factor beta, TLR4—Toll-like receptor 4, TREM2—triggering receptor expressed on myeloid cells 2, and tumor necrosis factor receptor—TNFR.

**Table 2:** Cellular effectors in COVID-19 lung fibrosis.

Cell Type	Role in Lung Fibrosis
Macrophages	present the antigens to pre-existing cross-reactive memory T cells, creating an activation loop between macrophages and T cells [38,39] activation of monocytes to MoAMs which express elevated levels of profibrotic genes such as *CD163, MERTK, LGMN, MMP9, TGF-β, NRP1, TGF-β1*, and *MRC1* [1] promotes lung fibrosis by interacting with mesenchymal cells such as fibroblasts, myofibroblasts, smooth muscle cells, and pericytes [1]
Epithelial alveolar cells	initiate lung inflammation that leads to fibrosis [48] are key components of tissue remodelling through re-epithelization processes [48] viral-induced necroptosis in epithelial alveolar cells (both type I and type II) leads to marked alveolar tissue architecture changes characterized by fibrosis and impaired re-epithelization [48] the induction of a senescence state in the alveolar cells type II (evidenced by an increased p21 and p16 nuclear expression) makes them produce a large number of pro-inflammatory molecules, most importantly TGF-β and IL-8 (important for its interaction with neutrophils) [48,50]
Neutrophils	the formation of NETs stimulates the EMT, which further promotes fibrosis [50] alveolar damage alone is not sufficient to initiate fibrosis; it also requires the presence of NETs [50,52] inflammatory cytokines (e.g., IL-17) stimulate neutrophil degranulation, increase oxidative stress damage, and augment fibrotic changes [52]
Fibroblasts	pro-inflammatory cytokines trans-differentiate fibroblasts into myofibroblasts, with architectural changes and further production of ECM (Collagen I—mature/Collagen III—immature and Fibronectin) [53,54] the autophagy processes in fibroblasts and myofibroblasts are reduced by activation of the PI3K/AKT/mTOR pathway and inhibition of EF2K and p38 MAPK signaling, increasing fibrotic lung changes [58] SARS-CoV-2 presents a broad tropism for various receptors, including integrins α_v_β_3_ and α_v_β_6_, inducing the transdifferentiation of fibroblasts into myofibroblasts, and the EMT mediated by TGF-β1, thus triggering fibrogenesis when the virus is attached [59] the interaction with a more rigid matrix amplifies myofibroblast synthesis activity, establishing a reinforcing loop [60] pulmonary lipofibroblasts increase pulmonary fibrosis, particularly in diabetic or obese patients [65]

**Abbreviations:** ECM—extracellular matrix, EMT—epithelial–mesenchymal transformation, IL—interleukine, LGMN—legumain, MERTK—tyrosine-protein kinase Mer, monocyte driven alveolar macrophages—MoAMs, MRC1—Mannose Receptor C-Type 1, NETs—neutrophil extracellular traps, and TGF-β—transforming growth factor beta.

**Table 3:** Specific aspects of COVID-19 lung fibrosis.

SARS-CoV-2 activates pulmonary myofibroblasts through pathways such as IL-6/STAT3 or TGF-β/Smad, upregulating COL1A1 gene (encoding for collagen type I alpha 1 chain) in these cells [27]
SARS-CoV-2 creates an imbalance between STAT1 and STAT3 signaling pathways by inhibiting STAT1 while hyperactivating STAT3 [28]
SARS-CoV-2 pneumonia showed persistently elevated T cell levels, associated with high values of IFN γ, with activation of MoAMs, which express elevated levels of profibrotic genes (*CD163, MERTK, LGMN, MMP9, TGF-β, TGF-β1, NRP1*, and *MRC1*) [16]
SARS-CoV2 can induce in CD163/LGMN-Mφ macrophage population phenotypes which express genes with known involvement in the pathological sequela of fibrosis, such as *SPP1, TGF-β I, LGMN*, and *CCL18* [16]. CD163/LGMN-Mφ present a strong interaction with myofibroblasts, fibroblasts, and pericytes, implying important fibrotic pathways that involve Col, FGF (fibroblast growth factor), TGF-β1, and SPP1, among others [12]
CD163+ macrophages act as profibrotic triggers expressing a phenotype and genes involved in the synthesis of TGF-β, osteopontin, and legumain, stimulating the proliferation of fibroblastic and myofibroblastic populations, increasing the production of collagen in fibroblasts, inhibiting MMP 14, and influencing the extracellular matrix composition, increasing the synthesis of collagen I [16,19].
The proportion of alternatively activated macrophages (AAM) associated with pulmonary fibrosis is substantially increased in COVID-19 patients [24,25]
SARS-CoV-2 induces a senescence state in the AT2 cells (evidenced by an increased p21 and p16 nuclear expression), increasing the production of pro-inflammatory molecules involved in pulmonary fibrosis [55,57]
SARS-CoV-2 presents a broad tropism for various receptors, including integrins α_v_β_3_ and α_v_β_6_. Integrin α_v_β_6_ fosters the transdifferentiation of fibroblasts into myofibroblasts and the EMT mediated by TGF-β1, thus triggering fibrogenesis when the virus is attached [66,93]
After being infected by SARS-CoV-2, an increase in TGF-β1 and CTGF mRNA transcripts in alveolar epithelial cells was demonstrated, leading to lung fibrosis [92]
SARS-CoV-2 can induce pulmonary fibrosis through its nucleocapsid (N) protein, shown to induce a heightened expression of α-SMA levels in HFL-1 (human fibroblast line)-type cells; thus, these cells incline towards more myofibroblast-like expression [94]
SARS-CoV-2 could activate fibroblasts in a cell-autonomous manner, potentially leading to lung fibrosis [94]
SARS-CoV-2 raises FN (fibronectin) 1 gene expression, indicating an incipient mean by which the virus might drive the development of lung fibrosis [98,99]

## Data Availability

The data presented in this study are available on request from the corresponding authors.
